# Prevalence and Triggering Factors of Childhood Anemia: An Application of Ordinal Logistic Regression Model

**DOI:** 10.1155/2022/2212624

**Published:** 2022-02-04

**Authors:** Md. Akhtarul Islam, Sohani Afroja, Md. Salauddin Khan, Sharlene Alauddin, Mst. Tanmin Nahar, Ashis Talukder

**Affiliations:** Statistics Discipline, Science Engineering and Technology School, Khulna University, Khulna 9208, Bangladesh

## Abstract

**Introduction:**

Anemia is indeed a significant risk factor for children's health as it affects growth retardation and has severe short and prolonged effects that follow in morbidity and death. Notwithstanding such ways to tackle anemia, the prevalence remains high in India and poses a severe public health concern.

**Objectives:**

The primary focus of this study was to find the prevalence and to determine the factors associated with the anemia of children under five years of age in India. *Problem Statement*. The increasing prevalence of childhood anemia and the life-threatening consequences for millions of children in India are a major concern. Knowing the relevant associated factors with childhood anemia is essential to reduce the frequency and severity level. *Study design*. For analysis purposes, this study utilized a cross-sectional study design. *Methodology*. Using the Indian Demographic and Health Survey 2015–16 data, we used chi-squared and gamma tests to find the association. Then, we utilized multinomial logistic regression and ordinal logistic regression to find the better model and the influencing factors of anemia in India.

**Results:**

In our study, we have found that children with highly educated mothers were 36.7% less likely (OR = 0.633, *P* ≤ 0.001, 95% CI: 0.608, 0.658) to be higher anemic than the children with not educated mother. Children with moderate and severe anemic mothers were 163.3% (OR = 2.633, *P* ≤ 0.001, 95% CI: 2.565, 7.704) more likely to be higher anemic than the children with not anemic mother. Not stunting children were 21.9% (OR = 0.781, *P* ≤ 0.001, 95% CI: 0 .764, 0.797) less likely to be higher anemic than the stunting children. Children aged 36–59 months were 73.9% (OR = 0.361, *P* ≤ 0.001, 95% CI: 0.353, 0.369) less likely to be higher anemic than the children aged 6–24 months. Again, the ACI value revealed that ordinal logistic regression was a better-fitted model for these data.

**Conclusion:**

and contribution. The variables such as stunting, underweight, wasting, child age, size of the child, and source of drinking water were the most critical indicators for child anemia in India. In summary, our study result indicated the major socioeconomic and demographic factors associated with childhood anemia in India, which can help the policymaker to take quick decision to reduce the severity level.

## 1. Introduction

The most salient nutritional disorder the developing countries are facing is childhood anemia, which is one of the major public health issues around the world [1, 2]. It is especially predominant in developing countries such as Africa and Asia, mainly Southeast Asia, where mostly one of every four individuals is affected by anemia; in particular, pregnant women and preschool-aged children are at the most serious risk [3]. The World Health Organization (WHO) declared that Africa and Southeast Asia have the most elevated risk. Around 66% of preschool-aged children and half of all women suffered from anemia [4].

According to WHO, “Anemia is a condition in which the amount of red blood cells or oxygen-carrying capacity of hemoglobin is insufficient to meet physiologic needs, which vary by age, sex, altitude, smoking, and pregnancy status.” Particularly, lack of iron in the body reduces the amount of hemoglobin, and the protein that transfers oxygen throughout the tissues of the body causes anemia. Although there are other physiological malfunctions such as thalassemia, hormone disorders, autoimmune disorders, vitamin B12 and vitamin A deficiencies, and inherited disorders, they can also induce anemia [5, 6].

The etiology of the anemic condition is multifactorial, and recent data suggest that less than 50% of people suffered from anemia because of iron deficiency [7]. In India, the diet mainly has a low iron concentration, and shreds of evidence revealed that low iron stock up depends on scattered surveys. It could be logical that most of the anemic condition is caused by iron deficiency [8–10]. To fight iron deficiency, the National Nutritional Anemia Prophylaxis Programme (NNAPP) was instigated in India [11]. Although the amount of iron and hemoglobin is increasing in the body and the occurrence of anemia is diminishing worldwide at a slower alacrity, there is also a reverse circumstance in South Asia and Central and West Africa where this disease is advancing as a burden day by day [12].

Anemia can affect people of all ages, races, and ethnicities such as children, pregnant and nonpregnant women, men, and elderly people worldwide, but it mostly affects children [13]. Feeling tired or weak, poor ability to work, extreme fatigue, pale skin, chest pain, fast heartbeat or shortness of breath, headache, dizziness or light-headedness, cold hands and feet, inflammation or soreness of your tongue, etc., can be the symptoms for anemia, and sometimes, it causes the death of the newly born child or premature birth or hampers the growth of the fetus [14]. It can double the risk of death during pregnancy and leads to poor physical and mental growth in children [15].

In India, the declining level regarding the prevalence of anemia has not been significant over the past five decades [16]. Globally, India bears the major overload of anemia, specifically in the case of women and infants [17–19]. Almost 50% of Indian women who are in their reproductive age are anemic as declared by National Family Health Survey in 2019 [20]. Women of reproductive age who have to go through anemia are exactly 41.5%, along with 3.0% more women who had faced severe anemia in India [21]. The pervasiveness of anemia among pregnant women still varies from 33% to 89% in diverse states of the nation [22]. In addition, low birthweight reduced cognitive growth, and poor immunity was identified in the newly born infant of anemic mothers in India [23].

Moreover, anemia remains a prevalent public health issue with a frequency of more than 50% among children and adolescents in India [16]. A study of the prevalence of anemia among adolescents in selected schools in India revealed that more than 50% of adolescents were anemic [24]. In that study, they had enrolled 499 adolescent students, and among them, 458 students had anemia. The data also exposed that the prevalence of anemia was 62.7 [24]. In India, the 2005–2006 National Family Health Survey (NFHS) revealed that children under five years old were 69.5% anemic [25]. Also, the 2015–2016 Demographic Health Survey revealed that 53.2% of nonpregnant women and 50.4% of pregnant women were anemic. Though the percentage of child anemia decreased by only 11% and became 58.5%, it is still an immense problem in India [26–28].

Furthermore, many researchers have utilized binary logistic regression [29–32], multilevel logistic regression [33, 34], and linear regression [35], and others have used a Gaussian model [27, 36, 37] to study variables that impact childhood anemia, which is perhaps the most prevalent approach used in certain studies. Researchers utilize a binary logistic model to analyze the child anemia as a binary predictor variable, and when evaluating the pediatric hemoglobin Hb level as a continuous response, a Gaussian model is applied. Yet, the focus on identifying the severity of disease and the risk factors that are associated with this is essential for epidemiological reasons [38, 39]. In cases, a similar study of multinomial ordered model has been published where nonanemia (Hb 11 g/dL), mild anemia (10.9 g/dL Hb 10 g/dL), moderate anemia (9.9 g/dL Hb 7.0 g/dL), and severe anemia (Hb 7.0 g/dL) might all be considered ordered responses in children [39]. To our experience, the use of ordinal logistic models for child anemia in India is rare. When resources are few, using this ordinal logistic model to identify children at the highest risk of anemia might be beneficial.

To reduce anemia in India, it is imperative to find out the influencing factors that upsurge the prevalence of anemia. Several studies have shown a comprehensible association between anemia and a list of determinants such as place of residence, states in India, educational status of parents, food habit, water facilities, toilet facilities, age of the child, birth order, and underweight [27, 28, 39–41]. This study aimed to assess the severity level of anemia and investigate the socioeconomic and demographic elements connected with childhood anemia. This study also focused on revealing a better-fitted model for the data. Moreover, this research can help public health policymakers to determine priorities for invention for reducing the anemia severity level.

## 2. Materials and Methods

### 2.1. Data Sources

This study utilized the most recent (round VII) Demographic and Health Survey (DHS) performed in 2015∼2016, also comparable to the National Family Health Survey (NHFS) (round 4) in India [26]. Additionally, in the NFHS-4 of the twenty-nine states in India, all seven union territories were inspected for the first time. As per the 2011 Census (IIPS, 2017; NFHS-4, 2009), this permitted the evaluation of many indicators for all 640 districts at the district level in India. The complete data set is accessible upon request from https://dhsprogram.com/and includes no recognizable evidence on the study participants. However, DHS is standard data that follow a nationally representative sampling procedure, objective measurement of anthropometric measures, accumulation of a wide range of monitoring and impact assessment statistics for health and nutrition, and high-ranking reply to rates (NFHS-4, 2009). After removing the missing data, we have added a total of 186123 respondents to this study.

### 2.2. Variables

In our study, the outcome variable was the anemic status of children aged 6 to 59 months, categorized into three outcomes as 1. not anemic, 2. mild, and 3. severe or moderate. In our research, we have used the explanatory variables at the individual and household levels. These include the following: place of residence was categorized as urban and rural; mother's educational level was categorized into four outcomes as no education, primary, secondary, and higher; sex of household head was tagged as male and female; sex of child was classified into two outcomes as male and female; mother's anemia level was categorized into three outcomes as 1. not anemic, 2. mild, and 3. moderate and severe; mother BMI was classified into three outcomes as normal, underweight, and overweight; and religion was defined in three outcomes as Hindu, Muslim, and others. We included the recategorized variables, and those are as follows: wealth index (recoded into three outcomes: poor, middle, and rich), mother's age at 1st birth (recoded into three outcomes: less than or equal to 18, 18–24, and greater than 24), child age (recoded into three outcomes: 6–24 months, 24–36 months, and 36–59 months), size of the child (recoded into three outcomes: average, small, and large), and birth order (also recoded into two outcomes: 1st and 2nd). We also included place of delivery (home and hospital), stunting (yes (if the children were stunted and no (otherwise)), underweight (yes (if the children having underweight) and no (otherwise)), wasting (yes (if the children having wasted) and no (otherwise)), breastfeeding status (no (if there was no record of breastfeeding history of the child) and yes (if there was record of breastfeeding history of the child)), had diarrhea recently (no (if there was no record of diarrhea recently) and yes (if there was record of diarrhea recently)), had fever in last two weeks( no (if the children had no fever in the previous two weeks) and yes (otherwise)), toilet facility (improved and not improved), and source of drinking water (improved and unimproved).

### 2.3. Logistic Regression Model

An ordinal logistic regression model was implemented because the child's anemia status is recorded. Notably, we applied the proportional odds model due to the reason of the subsequent attractive peculiarities: (a) it is invariant based on the numerous classes as if only the signs of the coefficients of regression differ when the coding of the explanatory variables is inverted; (b) it is also invariant under collapsibility of the assigned classes as the coefficient of the regression does not differ when the response classes are collapsed, or the class definitions are changed; and (c) it provides the explainable and straightforward coefficients of the regression as exp(−*β*) is the similar odds ratio (OR) over all cut-off points summarizing the effects of the explanatory variables on the response variable in a single frequently used measure [42–44]. The proportional odds model for the catagorical variable *Y* with *C* ordered categories for the *l*th subject with *P* independent variables. *X*_*l* _= (*x*_*il*_, *x*_2*l*_,   …, *x*_*pl*_), *l*=1,2,…, *n*, is given as follows:

(1) $$\begin{array}{c} \mathrm{logit}\;\left[Y_{l}\leq i|x_{l}\right]=\text{Log}\left[\frac{\pi_{i}\left(X_{l}\right)}{1-\pi_{i}\left(X_{l}\right)}\right]\\ =\alpha_{i}-\beta_{1}x_{1l}-\cdots -\beta_{p}x_{pl}\\ =\alpha_{i}-X_{l}^{/}\beta \;\mathrm{for}\;i=1,2,3,\ldots ,c-1;1=1,2,\ldots ,n, \end{array}$$

where *π*_*i*_(*X*_*l*_) = Pr[*Y*_*l* _ ≤ *i*|*x*_*l* _], *β* is a column vector of *P* coefficient of the regression, and *α*_*i*_ is the *i*th intercept. This study also used the multinomial logistic regression model to compare the results with the ordinal logistic regression model [45].

## 3. Results

The bivariate analyses of this study are reported in Table 1. From the table, it is vibrant that mother's education level, place of residence, sex of household head, toilet facility, breastfeeding status, and sex of child had fever in last two weeks, and mother's anemia level, religion, wealth index, mother's age at first birth, birth order, place of delivery, stunting, wasting, underweight, child age, size of child, and source of drinking water have a significant association with child anemia. The gamma estimates show that there exists a significant weak negative association with mother's education level, wealth index, mother's age at first birth, and child age and significant weak positive association with mother's anemia level and size of the child. No strong evidence of association is observed for mother's body mass index (BMI).

From Table 2, we can say that the probability of children being moderate and severe anemic in the rural area was 4.4% less (OR = 0.956, *P* ≤ 0.004, 95% CI: 0.927, 0.986) than the children from the urban area. Children with high educated mothers were 25.3% less (OR = 0.747, *P* ≤ 0.001, 95% CI: 0.71, 0.785) likely to be mild anemic compared with not anemic than the children with not educated mother. Children with higher educated mothers were 45.9% (OR = 0.541, *P* ≤ 0.001, 95% CI: 0.531, 0.571) less likely to be moderate and severe anemic compared with not anemic than the children with not educated mother. The probability of children being mild anemic from not anemic with unimproved toilet facility was 14.9% more (OR = 1.149, *P* ≤ 0.001, 95% CI: 1.114, 1.185) than the children with improved toilet facility. The probability of children being moderate and severe anemic from not anemic with unimproved toilet facility was 26.4% more (OR = 1.264, *P* ≤ 0.001, 95% CI: 1.225, 1.304) than the children with improved toilet facility. Children with moderate and severe anemic mother were 91% (OR = 1.908, *P* ≤ 0.001, 95% CI: 1.84, 1.979) more likely to be mild anemic and 251.2% (OR = 3.512, *P* ≤ 0.001, 95% CI: 3.392, 3.636) more likely to be moderate and mild anemic than the children with not anemic mother. The chance of being anemic of children with a mother's age at first birth less than 24 is less than the children with mother's age at first birth less than 18. Not stunting child were 13.1% (OR = 0.869, *P* ≤ 0.001, 95% CI: 0.845, 0.894) less likely to be anemic and 28% less likely to be (OR = 0.72, *P* ≤ 0.001, 95% CI: 0.70, 0.74) moderate and severe anemic than not anemic compared with stunting children. Average weight children were 11.6% less likely (OR = 0.884, *P* ≤ 0.001, 95% CI: 0.857, 0.912) to be mild anemic and 22.2% less likely (OR = 0.778, *P* ≤ 0.001, 95% CI: 0.754, 0.803) to be moderate and severe anemic than not anemic compared with underweight children. Not wasting children were 12.1% (OR = 0.879, *P* ≤ 0.001, 95% CI: 0.85, 0.908) less likely to be mild anemic and less likely to be (OR = 0.954, *P* ≤ 0.006, 95% CI: 0.923, 0.986) moderate and severe anemic than not anemic compared with wasting children. Children aged 36–59 were 45.1% less likely (OR = 0.549, *P* ≤ 0.001, 95% CI: 0.533, 0.565) to be mild anemic and 84.2% less (OR = 0.258, *P* ≤ 0.006, 95% CI: 0.251, 0.266) likely to be moderate and severe anemic than not anemic compared with children aged 6–24. 

In Table 3, the chance of children being higher anemic was 3.2% (OR = 0.968; 95% CI: 0.945–0.991; *P* ≤ 0.006) lower for the children who are from rural areas compared with those who are from urban areas. This study revealed that children with highly educated mothers were 36.7% less likely (OR = 0.633, *P* ≤ 0.001, 95% CI: 0.608, 0.658) to be higher anemic than the children with not educated mothers. From the table, it is also observed that children whose mothers play as a head of the family were 3.3% (OR = 0.967; 95% CI: 0.941–0.993; *P* ≤ 0.014) less likely of falling under severely anemic status compared with those whose fathers play as a head of the family. Children suffering from fever were 1.082 (OR = 1.082; 95% CI: 1.055–1.111; *P* ≤ 0.001) times more likely to be at severe anemia than healthy children. The chance of being at severe anemic status was 1.196 (OR = 1.196; 95% CI: 1.168–1.225; *P* ≤ 0.001) times higher for those children who have nonimproved toilet facilities than those having improved one. Children with moderate and severe anemic mother were 163.3% (OR = 2.633; 95% CI: 2.565–2.704; *P* ≤ 0.001) more likely to be severely anemic compared with the children with not anemic mother and 66.2% (OR = 1.662; 95% CI: 1.631–1.694; *P* ≤ 0.001) more likely to be mild anemic than the children with not anemia mother. The chance of children being severely anemic with mother's age greater than 24 years is decreased by 0.952 (OR = 0.952; 95% CI: 0.923–0.981; *P* ≤ 0.01) times compared with the children with mother's age at first birth less than 18.

From the AIC, it is clear that the ordinal logistic model is the better-fitted model for identifying the factors associated with child anemia in India.

## 4. Discussion

This research work detects some significant risk factors to build up different fundamental planning and implementing programs to reduce the prevalence of childhood anemia [46]. Our analysis reveals that the variable, type of residence, mother's education status, sex of household head, wealth index, mother's anemia level, stunting, underweight, wasting, and child age are significantly associated with the occurrence of childhood anemia in India. According to this research study, children from rural areas have a lower risk of anemia compared with urban areas. People living in rural areas most probably have poor knowledge of awareness and infants' food requirements. In addition, they are far away from the continuous change in lifestyle through the changes in increasing modernization and industrialization like urban areas [47–49]. This could be a possible reason for the increasing rate of anemia in rural communities. This analysis points out the worse situation of different socioeconomic, health, and nutrition factors in rural areas, and this panorama would be helpful in planning effective measures for controlling and maintaining government programs of the country [50]. The research study reveals that children of highly educated mothers were consistently less likely to be anemic than children who have illiterate mothers. An increasing level of education creates a better nutritional understanding and awareness about optimum child health. Some previous works of literature support these findings [47, 51]. Therefore, an increasing level of education enhances the quality of child health care and builds up a potential attention for preventing childhood anemia. This study identified mother's education as a significant effect modifier in influencing child's health conditions such as childhood anemia. A literate mother with their knowledge on nutritional education could contribute to the household well-being [36]. Perhaps some previous literature contradicted and found negative association between maternal education and children's nutritional status [52, 53]. Children living in a family with a female household head have a lower risk of being anemic. Although it is a significant factor, no other previous studies explore its effectiveness. The result of this study confirmed that the prevalence of anemia differed by a number of unnoticed key variables such as household head of a family [47]. Fever is often considered as one of the vital symptoms of acute and chronic diseases, including anemia [48]. Children having fever for two weeks are seemed to have a higher tendency of being anemic. This result satisfies some previous studies [47, 48]. It highlights the fact that fever is mostly accompanied by other morbidities such as diarrhea and malaria, which also positively affect childhood anemia [36]. Maternal anemia is significantly associated with the occurrence of childhood anemia. Children will have a high risk of anemia if their mothers are already suffering from anemia, which supported some previous studies [1, 54–57]. Several maternal infections, immunization, and dietary conditions could reflect the anemic status of children. In addition, most children share a common dietary intake and socioeconomic environment with their mother, which indicates that mother's health status has a significant contribution to the occurrence of childhood anemia. According to this study, both the maternal anemia and child anemia reflect a common nutritional status of the household. So, healthcare policies could gain beneficiary outcome in reducing childhood anemia by emphasizing this factor [58]. Religious affiliations significantly contribute to the child's anemia status. Muslim children have a higher tendency to be anemic, while others have a lower tendency than Hindu children. The finding of this study is supported by some other previous related literature, which is conducted in different regions [27, 38]. Increasing order in birth tends to a higher risk of being anemic among children aged between 6 and 59 months. This might happen because parents provide less attention to their children if they have many of them. Some literature still points out this factor as insignificant [41]. The result demonstrated that nonstunted children have a lower tendency to be anemic than the stunted children. This finding demonstrates that stunting is significantly associated with the up growing risk of anemia among the children aged between 6 and 59 months who are stunted [47, 59]. This finding of the research analysis will be helpful to underpin cost‐effective programming and to take obligate actions to reduce the prevalence of childhood anemia [60]. A significant diminishing risk of anemia is found among the children who are neither underweight nor wasted. Children's nutritional status could be defined in terms of stunting, wasting, and underweight. According to this research work, undernourished children are experiencing a higher risk of increasing anemia than nourished children [61]. The correlation between physical growth and anemia is documented in previous studies [62, 63]. This research emphasized the importance of healthy weight to create awareness and prevent the occurrence of anemia. This study indicates that children with the age range of 24–36 months and 36–59 months had a lower tendency to be anemic than the children with the age range of 6–24 months. The finding of this study is consistent with the result of some previous literature [39, 41, 64]. Child size at birth is found to be a significant reflecting factor in the existence of anemia. Children who are large in size have a lower tendency to be anemic, while on the other hand small size children have a greater risk of anemia. So, early care should be taken during the time of pregnancy. Some previous literature opposes these findings and could not find it as a significant factor [41, 47]. Evidence shows that children whose households are not facilitated with the improved source of drinking water have a higher risk of anemia. In most developing countries, water contaminated by iron and polluted particles could be responsible for the causation of children's anemia [65]. The role of contaminated water supply is consistent with several previous findings [33, 66]. This study demonstrates that the increased odds of being anemic are found among the children whose mothers are underweight and it is also true for the mothers who gave birth to their first child at the age between 18 and 24 years. On the other hand, women who enjoy their first motherhood at the age of 24 and are being overweight have a comparatively lower risk of anemia among their children [39]. In this analysis, children from poor family backgrounds have a lower tendency to be anemic compared with the children of rich family backgrounds in India, which is a contradiction of different existing studies [67–69]. This study established a link between the indicators of socioeconomic status and risk of anemia. The findings will amplify the need for targeted efforts to focus on the children belonging to poor families.

This study has some limitations, as it utilized cross-sectional data and could not include and address the previous year's survey data and information. It was also unable to address and include some other important variables related to child anemia because of missing information or unavailability of the variables in the data set. The strong sides of this study are the utilization of the updated Indian Demographic and Health Survey data to show the prevalence of different selected variables and the application of two different models to find the associated factors of child anemia in India. This study also showed a better-fitted model between two utilized models for this data set which was another strong point. 

## 5. Conclusion

This research study highlights the prevalence of anemia as a severe public health challenge in India. A comparative analysis has been conducted in this study using the multinomial and ordinal logistic regression model. The ordinal logistic regression model was found to be better-fitted in identifying the effect of influencing factors of anemia among children aged 6–59 months. In the ordinal logistic regression model, we found that maternal education, stunting, underweight, wasting, child age, size of the child, and source of drinking water are significantly impactful determinants of anemia among children aged 6–59 months. This study is emphasizing higher maternal education, improved water, and toilet facilities, especially in rural areas. The findings of this study will contribute to recommending the government and policymakers of India to build up impactful policies and design proper interventions that could significantly prevent anemia among children aged 6–59 months.

## Figures and Tables

**Table 1:** Baseline characteristics for selected determinants causing anemia among children aged 6–59 months in India.

Variables	Measurement scale	Categories	Child anemic status			*χ*2 (*P* value)	Gamma (*P* value)
			Not anemic % (*n*)	Mild % (*n*)	Severe or moderate % (*n*)		
Place of residence	Nominal	Urban	45.4 (20365)	26.2 (11744)	28.5 (12785)	147.449 **(P** ≤ **0.001)**	
		Rural	42.1 (59499)	27.4 (38693)	30.5 (43037)		

Mother's educational level	Ordinal	No education	35.9 (20827)	28.2 (16357)	35.9 (20870)		−0.150 (*P* ≤ 0.001)
		Primary	42 (11356)	27.3 (7380)	30.7 (8291)		
		Secondary	46.5 (39198)	26.5 (22326)	27 (22769)		
		Higher	50.6 (8483)	26.1 (4374)	23.2 (3892)		

Sex of household head	Nominal	Male	42.9 (70471)	27 (44328)	30.1 (49452)	12.564 (0.002)^*∗*^	
		Female	42.9 (9393)	27.9 (6109)	29.1 (6370)		

Breastfeeding status	Nominal	No	48.3 (33481)	26.3 (18244)	25.5 (17665)	1520.637 **(P** ≤ **0.001)**	
		Yes	39.7 (46383)	27.6 (32193)	32.7 (38157)		

Sex of child	Nominal	Male	43 (41557)	26.7 (25840)	30.2 (29206)	13.531 **(P** ≤ **0.001)**	
		Female	42.8 (38307)	27.5 (24597)	29.7 (26616)		

Had diarrhea recently	Nominal	No	43.6 (73812)	27.2 (46026)	29.2 (49527)	554.350 **(P** ≤ **0.001)**	
		Yes	36.1 (6052)	26.3 (4411)	37.6 (6295)		

Had fever in last two weeks	Nominal	No	43.4 (70100)	27.3 (44067)	29.3 (47327)	278.130 **(P** ≤ **0.001)**	
		Yes	39.6 (9764)	25.9 (6370)	34.5 (8495)		

Toilet facility	Nominal	Improved	47.4 (44981)	26 (24661)	26.6 (25243)	1740.712 **(P** ≤ **0.001)**	
		Not improved	38.2 (34883)	28.3 (25776)	33.5 (30579)		

Mother's anemia level	Ordinal	Not anemic	52.3 (43280)	25.2 (20858)	22.5 (18571)		0.279 (*P* ≤ 0.001)
		Mild	38 (28743)	29.2 (22080)	32.8 (24824)		
		Moderate and severe	28.2 (7841)	27 (7499)	44.8 (12427)		

Religion	Nominal	Hindu	41.1 (55609)	27.8 (37710)	31.1 (42100)	2296.820 (*P* ≤ 0.001)	
		Muslim	40.6 (11672)	26.7 (7684)	32.7 (9423)		
		Other	57.4 (12583)	23.0 (5043)	19.6 (4299)		

Wealth index	Ordinal	Poor	39.8 (36411)	27.9 (25583)	32.3 (29539)		−0.095 **(P** ≤ **0.001)**
		Middle	44.2 (16628)	26.6 (9993)	29.2 (10987)		
		Rich	47.1 (26825)	26.1 (14861)	26.8 (15296)		

Mother's age at 1st birth	Ordinal	Less than or equal to 18	41.9 (17513)	27.9 (11648)	30.2 (12611)		−0.046 **(P** ≤ **0.001)**
		18–24	42.0 (48516)	27.3 (31512)	30.7 (35529)		
		Greater than 24	48.0 (13835)	25.3 (7277)	26.7 (7682)		

Birth order	Ordinal	1st	45.3 (30587)	27.3 (18418)	27.4 (18511)		0.070 (*P* ≤ 0.001)
		2nd	43 (24913)	27.1 (15682)	29.9 (17333)		
		Other	40.2 (24364)	26.9 (16337)	32.9 (19978)		

Place of delivery	Nominal	Home	42.2 (18454)	27 (11787)	30.8 (13438)	17.447 **(P** ≤ **0.001)**	
		Hospital	43.1 (61410)	27.1 (38650)	29.8 (42384)		

Stunting	Nominal	Yes	37.5 (28243)	27.4 (20670)	35.1 (26458)	1981.169 **(P** ≤ **0.001)**	
		No	46.6 (51621)	26.9 (29767)	26.5 (29364)		

Underweight	Nominal	Yes	36.4 (24231)	28.2 (18762)	35.4 (23582)	2088.298 **(P** ≤ **0.001)**	
		No	46.5 (55633)	26.5 (31675)	27 (32240)		

Wasting	Nominal	Yes	37.6 (13825)	29.2 (10736)	33.1 (12173)	524.156 **(P** ≤ **0.001)**	
		No	44.2 (66039)	26.6 (39701)	29.2 (43649)		

Child age	Ordinal	6–24 months	31.4 (19829)	27.3 (17236)	41.3 (26086)		−0.299 **(P** ≤ **0.001)**
		24–36 months	40.7 (16857)	27.6 (11445)	31.7 (13124)		
		36–59 months	52.9 (43178)	26.7 (21756)	20.4 (16612)		

Size of child	Ordinal	Average	43.2 (57012)	27.1 (35740)	29.8 (39369)		0.006 **(P** ≤ **0.001)**
		Small	40.3 (8861)	26.4 (5808)	33.3 (7313)		
		Large	43.7 (13991)	27.8 (8889)	28.5 (9140)		

Source of drinking water	Nominal	Improved	44.6 (31049)	26.3 (18351)	29.1 (20268)	124.987 (*P* ≤ 0.001)	
		Unimproved	41.9 (48815)	27.6 (32086)	30.5 (35554)		

Mother BMI	Ordinal	Normal	43.5 (49881)	27.2 (31196)	29.2 (33513)		0.003 (0.330)
		Underweight	37.5 (16972)	28 (12701)	34.5 (15645)		
		Overweight	49.6 (13011)	24.9 (6540)	25.4 (6664)		

**Table 2:** Illustrating various socioeconomic and demographic factors influencing anemia among children aged 6 to 59 months in India using multinomial logistic regression.

Variable	Categories	Odds ratio		*P* value		95% confidence interval			
		Mild vs. not anemic	Moderate and severe vs. not anemic	Mild vs. not anemic	Moderate and severe vs. not anemic	Mild vs. not anemic		Moderate and severe vs. not anemic	
						Upper	Lower	Upper	Lower
Place of residence	Urban (ref)	—	—	—	—	—	—	—	—
	Rural	0.993	0.956	0.673	**0.004** ^*∗*^	0.964	1.024	0.927	0.986

Mother's educational level	No education (ref)	—	—	—	—	—	—	—	—
	Primary	0.871	0.785	**P** ≤ **0.001**	**P** ≤ **0.001**	0.84	0.904	0.756	0.814
	Secondary	0.78	0.641	**P** ≤ **0.001**	**P** ≤ **0.001**	0.756	0.804	0.621	0.661
	Higher	0.747	0.541	**P** ≤ **0.001**	**P** ≤ **0.001**	0.71	0.785	0.513	0.571

Sex of household head	Male (ref)	—	—	—	—	—	—	—	—
	Female	1.028	0.952	0.126	**0.007** ^*∗*^	0.992	1.064	0.918	0.987

Breastfeeding status	No (ref)	—	—	—	—	—	—	—	—
	Yes	1.01	0.982	0.445	0.174	0.985	1.036	0.956	1.008

Sex of child	Male (ref)	—	—	—	—	—	—	—	—
	Female	1.034	0.991	**0.004**	0.46	1.01	1.057	0.969	1.014

Had fever in last two weeks	No (ref)	—	—	—	—	—	—	—	—
	Yes	0.976	1.113	0.167	**P** ≤ **0.001**	0.943	1.01	1.076	1.151

Toilet facility	Improved (ref)	—	—	—	—	—	—	—	—
	Nonimproved	1.149	1.264	**P** ≤ **0.001**	**P** ≤ **0.001**	1.114	1.185	1.225	1.304

Mother's anemia level	Not anemic (ref)	—	—	—	—	—	—	—	—
	Mild	1.534	1.913	*P* ≤ 0.001	*P* ≤ 0.001	1.497	1.572	1.865	1.962
	Moderate and severe	1.908	3.512	*P* ≤ 0.001	*P* ≤ 0.001	1.84	1.979	3.392	3.636

Religion	Hindu (ref)	—	—	—	—	—	—	—	—
	Muslim	0.987	1.051	0.447	**0.003** ^*∗*^	0.955	1.02	1.017	1.086
	Other	0.674	0.545	**P** ≤ **0.001**	**P** ≤ **0.001**	0.649	0.7	0.523	0.567

Wealth index	Poor (ref)	—	—	—		—	—	—	—
	Middle	1.027	1.102	0.121	**P** ≤ **0.001**	0.993	1.063	1.064	1.14
	Rich	1.084	1.207	**P** ≤ **0.001**	**P** ≤ **0.001**	1.042	1.128	1.16	1.257

Age at 1st birth	≤18 (ref)	—	—	—	—	—	—	—	—
	18–24	0.997	1.057	0.825	**P** ≤ **0.001**	0.969	1.026	1.027	1.088
	˃24	0.893	0.953	**P** ≤ **0.001**	**0.019** ^*∗*^	0.858	0.929	0.915	0.992

Birth order	1st (ref)	—	—	—	—	—	—	—	—
	2nd	1.003	1.069	0.856	**P** ≤ **0.001**	0.975	1.031	1.038	1.1
	Other	0.997	1.119	0.852	**P** ≤ **0.001**	0.967	1.028	1.085	1.154

Place of delivery	Home (ref)	—	—	—	—	—	—	—	—
	Hospital	1.033	1.03	0.027	**0.045** ^*∗*^	1.004	1.064	1.001	1.061

Stunting	Yes (ref)	—	—	—	—	—	—	—	—
	No	0.869	0.72	**P** ≤ **0.001**	**P** ≤ **0.001**	0.845	0.894	0.7	0.74

Underweight	Yes (ref)	—	—	—	—	—	—	—	—
	No	0.884	0.778	**P** ≤ **0.001**	**P** ≤ **0.001**	0.857	0.912	0.754	0.803

Wasting	Yes (ref)	—	—	—	—	—	—	—	—
	No	0.879	0.954	**P** ≤ **0.001**	**0.006** ^*∗*^	0.85	0.908	0.923	0.986

Child age	6–24 months (ref)	—	—	—	—	—	—	—	—
	24–36 months	0.761	0.552	*P* ≤ 0.001	**P** ≤ **0.001**	0.736	0.786	0.535	0.569
	36–59 months	0.549	0.258	*P* ≤ 0.001	**P** ≤ **0.001**	0.533	0.565	0.251	0.266

Size of child	Average (ref)	—	—	—	—	—	—	—	—
	Small	0.988	1.074	0.508	**P** ≤ **0.001**	0.952	1.024	1.036	1.113
	Large	1.022	0.959	0.155	**0.008** ^*∗*^	0.992	1.054	0.929	0.989

Source of drinking water	Improved (ref)	—	—	—	—	—	—	—	—
	Unimproved	0.957	0.875	**P** ≤ **0.001**	**P** ≤ **0.001**	0.933	0.982	0.853	0.898

Mother BMI	Normal (ref)	—	—	—	—	—	—	—	—
	Underweight	1.032	1.089	0.026	**P** ≤ **0.001**	1.004	1.062	1.059	1.12
	Overweight	0.944	1.00	**P** ≤ **0.001**	0.997	0.912	0.978	0.965	1.037

AIC	377909.9								

**Table 3:** Illustrating various socioeconomic and demographic factors influencing anemia among children aged 6 to 59 months in India using ordinal logistic regression.

Variable	Categories	Odds ratio	*P* value	95% confidence	
				Lower	Upper
Place of residence	Urban (ref)	—	—	—	—
	Rural	0.968	**0.006** ^*∗*^	0.945	0.991

Mother's educational level	No education (ref)	—	—	—	—
	Primary	0.831	**P** ≤ **0.001**	0.808	0.855
	Secondary	0.713	**P** ≤ **0.001**	0.696	0.73
	Higher	0.633	**P** ≤ **0.001**	0.608	0.658

Sex of household head	Male (ref)	—	—	—	—
	Female	0.967	**0.014** ^*∗*^	0.941	0.993

Breastfeeding status	No (ref)	—	—	—	—
	Yes	0.989	0.256	0.969	1.008

Sex of child	Male (ref)	—	—	—	—
	Female	0.998	0.810	0.981	1.015

Had fever in last two weeks	No (ref)	—	—	—	—
	Yes	1.082	**P** ≤ **0.001**	1.055	1.111

Toilet facility	Improved (ref)	—	—	—	—
	Nonimproved	1.196	**P** ≤ **0.001**	1.168	1.225

Mother's anemia level	Not anemic (ref)	—	—	—	—
	Mild	1.662	**P** ≤ **0.001**	1.631	1.694
	Moderate and severe	2.633	**P** ≤ **0.001**	2.565	2.704

Religion	Hindu (ref)	—	—	—	—
	Muslim	1.039	**0.003** ^*∗*^	1.014	1.066
	Other	0.618	**P** ≤ **0.001**	0.6	0.636

Wealth index	Poor (ref)	—	—	—	—
	Middle	1.075	**P** ≤ **0.001**	1.048	1.104
	Rich	1.153	**P** ≤ **0.001**	1.118	1.189

Age at 1st birth	≤18 (ref)	—	—	—	—
	18–24	1.038	**0.001** ^*∗*^	1.016	1.061
	˃24	0.952	**0.002** ^*∗*^	0.923	0.981

Birth order	1st (ref)	—	—	—	—
	2nd	1.049	**P** ≤ **0.001**	1.026	1.071
	Other	1.082	**P** ≤ **0.001**	1.057	1.107

Place of delivery	Home (ref)	—	—	—	—
	Hospital	1.025	**0.030** ^*∗*^	1.002	1.048

Stunting	Yes (ref)	—	—	—	—
	No	0.781	**P** ≤ **0.001**	0.764	0.797

Underweight	Yes (ref)	—	—	—	—
	No	0.829	**P** ≤ **0.001**	0.809	0.848

Wasting	Yes (ref)	—	—	—	—
	No	0.952	**P** ≤ **0.001**	0.928	0.976

Child age	6–24 months (ref)	—	—	—	—
	24–36 months	0.632	**P** ≤ **0.001**	0.617	0.648
	36–59 months	0.361	**P** ≤ **0.001**	0.353	0.369

Size of child	Average (ref)	—	—	—	—
	Small	1.053	**P** ≤ **0.001**	1.024	1.082
	Large	0.975	**0.037** ^*∗*^	0.953	0.998

Source of drinking water	Improved (ref)	—	—	—	—
	Unimproved	0.905	**P** ≤ **0.001**	0.888	0.923

Mother BMI	Normal (ref)	—	**P** ≤ **0.001**	—	—
	Underweight	1.064	**P** ≤ **0.001**	1.042	1.087
	Overweight	0.984	0.247	0.958	1.011

AIC	**378309.8**				

## Data Availability

Data are available in the following website: https://dhsprogram.com/methodology/survey/survey-display-541.cfm.
